# Prognosis prediction and comparison between pancreatic signet ring cell carcinoma and pancreatic duct adenocarcinoma: a retrospective observational study

**DOI:** 10.3389/fendo.2023.1205594

**Published:** 2023-07-17

**Authors:** Hui Zhou, Xiao-xue Li, Yun-peng Huang, Yong-xiang Wang, Heng Zou, Li Xiong, Zhong-tao Liu, Yu Wen, Zi-jian Zhang

**Affiliations:** ^1^Department of General Surgery, Second Xiangya Hospital, Central South University, Changsha, Hunan, China; ^2^Department of Obstetrics and Gynecology, Second Xiangya Hospital, Central South University, Changsha, Hunan, China

**Keywords:** pancreatic signet ring cell carcinoma, pancreatic duct adenocarcinoma, pathologic subtype, risk score, prognosis

## Abstract

**Background:**

Pancreatic signet ring cell carcinoma (PSRCC) is a rare and aggressive cancer that has been reported primarily as case reports. Due to limited large-scale epidemiological and prognostic analyses, the outcomes of PSRCC patients varies greatly in the absence of recognized first-line treatment strategies. This study aimed to compare the clinical features, treatment, and prognosis of PSRCC and pancreatic ductal cell carcinoma (PDAC), the most common subtype of pancreatic cancer, and to establish predictive models for these subtypes.

**Methods:**

The data on PSRCC and PDAC patients from 1998 to 2018 was obtained from the Surveillance, Epidemiology, and End Results (SEER) database. Thereafter, the clinical, demographic, and treatment characteristics of the two groups and the differences and influencing factors of the two groups were evaluated by propensity score matching (PSM), Kaplan–Meier survival curves, Cox risk regression analyses, and least absolute shrinkage and selection operator (LASSO) analysis. Next, prognosis models were constructed and validated by KM and ROC analysis. Finally, a nomogram was constructed, based on the results of these analyses, to predict survival outcomes of PSRCC and PDAC patients.

**Results:**

A total of 84,789 patients (432 PSRCC and 84357 PDAC patients) were included in this study. The results of the study revealed that, compared to the PDAC patients, PSRCC patients were more likely to be male, aged between 58–72 years, have larger tumor masses, and less likely to undergo chemotherapy. Before PSM, the overall survival and cancer-specific survival of the PSRCC group were significantly lower than those PDAC group, but there was no difference in the prognosis of the two groups after PSM. Additionally, lymph node ratio (LNR), log odds of positive lymph node (LODDS), tumor size, age, T-stage, marital status, and summary stage were found to be independent prognostic factors for PSRCC. Lastly, the prediction model and nomogram based on these prognostic factors could accurately predict the survival rate of the patients in SEER datasets and external validation datasets.

**Conclusion:**

The prognosis of PSRCC and PDAC patients is similar under the same conditions; however, PSRCC patients may have more difficulty in receiving better treatment, thus resulting in their poor prognosis.

## 1 Introduction

Pancreatic cancer (PC) is a common highly aggressive malignancy of the digestive tract (1). Its increasing rates of morbidity and mortality and extremely poor prognosis make it one of the deadliest cancers worldwide (1, 2). PC has few clinical manifestations and often progresses to advanced stages before symptoms such as abdominal pain and abdominal masses appear, making its treatment very difficult. Although surgery, chemotherapy, radiotherapy, immunotherapy, and targeted therapy can be used to treat PC, these treatments are not always effective (3, 4). Although most PCs are classified as pancreatic ductal adenocarcinoma (PDAC), some rare pathological subtypes of PCs, such as pancreatic signet ring cell carcinoma (PSRCC), also exist (5). Although targeted therapy and immunotherapy have improved patient prognosis to an extent, several studies have reported that PC progression may be different in different pathological subtypes (6, 7). Furthermore, sensitivity to chemotherapy, radiotherapy, targeted therapy, and immunotherapy may vary in different PC subtypes. Therefore, it is extremely important to explore the prognostic differences and influencing factors of different PC subtypes to improve their diagnosis and prognosis.

PSRCC is a specific type of pancreatic mucus-secreting adenocarcinoma that originates from the undifferentiated stem cells in the lamina propria (8). The mucus secreted by the PSRCC cells is not discharged outside the cell, and the accumulating mucus squeezes the nucleus to the periphery of the cell, making the whole cell appear like a signet ring, with poor differentiation, diffused infiltration, rapid growth, high degree of malignancy, and high metastasis and recurrence (9). However, due to its low incidence, no predictive models have been reported for PSRCC and it is unclear whether there is a difference in prognosis between PSRCC and common PDACs. Considering the overall increase in PC incidence, high mortality, limited treatment options, poor prognosis, and short overall survival (OS), a few PSRCC studies suggest an urgent need for methods to calculate the survival probability of PSRCC patients based on different prognostic factors (10). The Surveillance, Epidemiology, and End Results (SEER) database contains population-based clinical survival data from registries covering 34.6% of the US population (11). In this study, we explored the prognostic differences between PSRCC and PDAC based on the SEER database statistics and developed a prognostic model and nomogram for prognostic prediction of PSRCC.

## 2 Materials and methods

### 2.1 Data source and case selection

Data on PSRCC and PDAC patients were extracted from the SEER database of the National Cancer Institute (http://www.seer.cancer.gov), released in November 2021 *via* SEER*Stat software (v8.4.0.1) (12). As the data in the SEER database were de-identified and coded for public availability, this study was exempted from the requirement to obtain approval from the Second Xiangya Hospital of Central South University Review Board.

**Figure 1:**
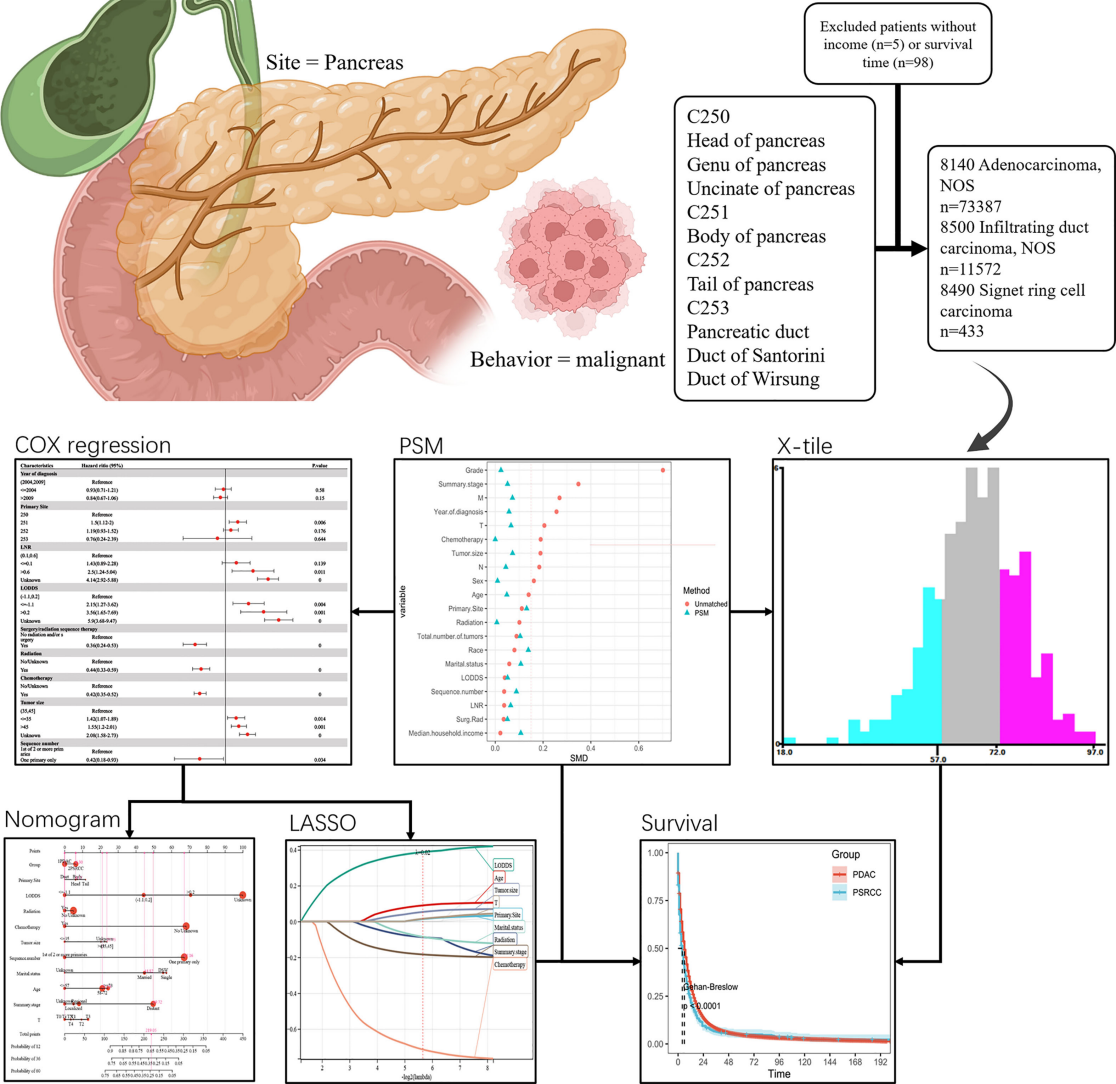
Flow chart of the study.

From 1998 to 2018, PC in the SEER program was identified *via* the site-specific International Classification of Oncological Diseases 3 (ICD-O-3) codes: C250, C251, C252, and C253. Although PC, as defined by SEER, may also include codes C254 and C257, these codes were not analyzed in this study. The diagnosis of PSRCC was determined using the ICD-O-3 codes 8490/3 (signet ring cell carcinoma, SRCC), while that of PDAC was determined using the ICD-O-3 codes 8140/3 (adenocarcinoma, NOS) and 8500/3 (infiltrating duct carcinoma, NOS). The number of primary tumors was identified using the sequence number for a single primary or the first of two or more primaries. The following cases were excluded from the study: (1) presence of non-primary tumor; (2) lack of complete follow-up data; and (3) lack of important data, such as household income. After the final screening, 84857 PDAC and 432 PSRCC patients were selected for this study. The codes for case collection complied with the guidelines of the SEER database coding and staging manual (**Figure 1**).

### 2.2 Clinical information acquisition

The following variables were obtained from the SEER database: demographic information (patient ID, sex, year of diagnosis, age at diagnosis, median household income, race, and marital status), tumor characteristics (histologic type; primary site; tumor size; total number of tumors; tumor grade; tumor, node, metastasis [TNM] stage; summary stage; sequence number; and lymph node ratio [LNR], and log odds of positive lymph node [LODDS]), treatment (surgery/radiation sequence therapy, radiation therapy, and chemotherapy), and follow-up for survival (survival months, cause-specific death, and vital status). Among these, LNR and LODDS, two novel staging systems for predicting lymph node metastasis, were further calculated from the data obtained from the SEER database. LNR is the ratio of positive lymph nodes to the retrieved lymph nodes, while LODDS is defined as log [(0.5 + positive lymph nodes)/(0.5 + negative lymph nodes)]. Since LNR and LODDS were identified as efficient prognostic factors for various malignant tumors, in this study, we further investigated their role in PDAC and PSRCC prognosis.

### 2.3 Preliminary data processing

For data processing, demographic information was recorded as sex (male or female), race (white, black, or other/unknown), and marital status (single, married, divorced/separated/widowed (DSW), or unknown). Tumor characteristics were recorded as histological type (PDAC or PSRCC), primary site (head, body, tail, or duct), total number of tumors (1–5), tumor grade (1, 2, 3, 4, or unknown), TNM stage (T-stage: T0/Ti/TX, T1, T2, T3, or T4; N-stage: N0, N1, N2, or NX; and M-stage: M0, M1, or MX), summary stage (distant, localized, regional, or unknown), sequence number (only one primary or first of 2 or more primaries). Lastly, treatment information was recorded as follows: surgery/radiation sequence therapy (yes or no radiation and/or surgery), chemotherapy (yes, no, or unknown), and radiation therapy (yes, no, or unknown).

**Figure 2:**
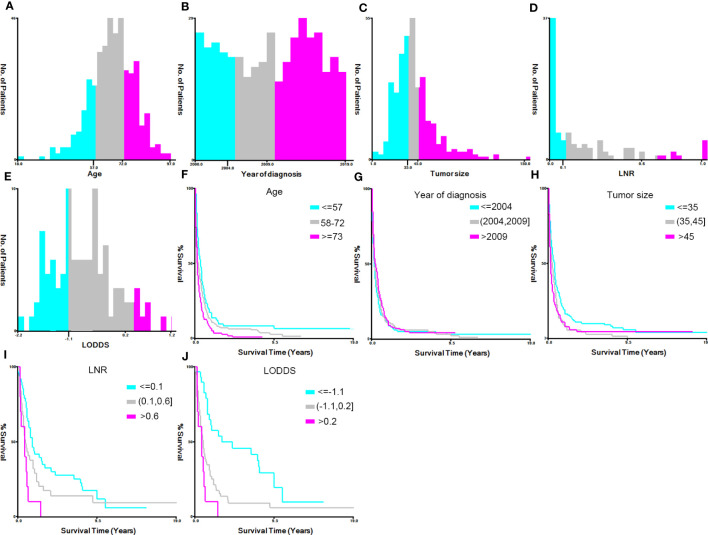
Identification of the optimal cutoff values for the variables ‘year of diagnosis’, ‘age’, ‘tumor size’, ‘lymph node ratio’ (LNR), and ‘log odds ratio’ (LODDS) *via* X-tile software analysis. **(A–E)** Histograms of patient distribution according to the year of diagnosis, age, tumor size, LNR, and LODDS, respectively. **(F–J)** The Kaplan–Meier curves of the year of diagnosis, age, tumor size, LNR, and LODDS of pancreatic signet ring cell carcinoma patients, respectively.

X-tile software (v3.6.1) was used to calculate the optimum cutoff value for converting the continuous variables (year of diagnosis, age at diagnosis, tumor size, LNR, and LODDS) into categorical variables. The variable ‘year of diagnosis’ was grouped as ‘≤2004’, ‘2004–2009’, and ‘>2009’; ‘age at diagnosis’ (years) was categorized into ‘≤57’, ‘57–72’, and ‘>73’; tumor size (mm) was grouped as ‘≤35’, ‘35–45’, ‘>45’, and ‘unknown’; LNR was grouped as ‘≤0.1’, ‘0.1–0.6’, ‘>0.6’, and ‘unknown’; and LODDS was categorized as ‘≤-1.1’, ‘-1.1–0.2’, ‘>0.2’, and ‘unknown’ (**Figure 2**).

### 2.4 Propensity score matching and survival analysis

As the Chi-square or Fisher’s exact tests revealed that the clinical characteristics of the PSRCC and PDAC cases in the SEER database were heterogeneous, we conducted PSM to adjust the baseline characteristics of the two groups. The following PSM settings were performed using the R package ‘MatchIt’ (v4.1.0): 1-to-1 pairing and nearest neighbor methods, with a caliper of 0.05 (13). The PSM included all the aforementioned variables. The OS and cancer-specific survival (CSS) were set as the outcome endpoints of the present study. The OS was defined as the time interval between diagnosis and death by any cause, while CSS was defined as the duration between diagnosis and death caused by cancer. The survival plot was constructed *via* Kaplan–Meier (KM) analysis, and the comparison between PSRCC and PDAC patients before and after PSM was conducted *via* logrank test.

### 2.5 COX regression analysis

Univariate and multivariate Cox regression analyses were conducted to determine the potential prognostic variables of the OS and CSS of PSRCC patients.

### 2.6 Least absolute shrinkage and selection operator regression analysis and visualization

COX regression analysis often has collinear interference. After PSM and Cox regression analysis, we used LASSO regression analyses to determine the optimal weighting coefficients of different pathological types and clinical characteristics and built a model to determine if these features can predict the prognosis of PSRCC and PDAC patients. LASSO regression models for the OS and CSS of PSRCC and PDAC patients were built by performing ten-fold cross-validation using the R package “glmnet” (14). Moreover, the optimal λ values of OS and CSS were 0.0195 and 0.0198, respectively.

Thereafter, the receiver operating characteristic (ROC) curve of the follow-up outcomes and risk scores over ten years was analyzed using the R package “pROC” and the area under the curve (AUC) and confidence interval (CI) was determined (15). Based on the optimal cutoff or median of risk scores, patients were categorized into high- and low-risk groups, and the prognostic differences between the two groups were further analyzed using the R package “survival”. The significant differences in the prognosis of the two groups were then assessed using the logrank test.

Moreover, to verify the risk score models externally, a total of 90 patients with PDAC or PSRCC as the external validation datasets from the Department of Hepatobiliary Surgery of the Second Xiangya Hospital were included in our study. According to the median cutoff values in the external validation datasets, patients were divided into high- and low-risk scores groups to verify the robustness of the model. KM analysis of OS or CSS was used to test the distinguishing effect between patients with high scores and those with low scores. Finally, ROC curves were used to evaluate the accuracy and predictive ability of the models in the external validation datasets.

Finally, survival data from the LASSO-COX analysis was integrated through the R package “rms” to construct nomograms and predict the 1-, 3-, and 5-year OS and CSS of PSRCC and PDAC patients. A nomogram calculates the risk of disease or an individual’s probability of survival by integrating multiple predictors and plotting multiple lines to scale and it uses the C-index to assess the power of the nomogram. In addition, the calibration curves of 1-, 3-, and 5-years were drawn to evaluate the effectiveness of the nomogram.

## 3 Results

### 3.1 Comparison of the baseline clinical characteristics of PSRCC and PDAC

A total of 84789 patients (432 PSRCC and 84357 PDAC patients) from the SEER database, were enrolled in this study (**Figure 1**). Several baseline clinical characteristics between PDAC and PSRCC groups were significantly different (*p <*0.05; **Table 1**). For instance, compared to the PDAC patients, PSRCC patients were more likely to be male (59.7% vs. 51.7%, *p* = 0.001) and aged between 58–72 years (50.5% vs. 44.7%, *p* = 0.018). Moreover, the PSRCC group had a lower percentage of ‘>2009’ group (49.3% vs. 60.5%) and a higher percentage of ‘≤2004’ group (26.9% vs. 17.4%) compared to the PDAC group (*p <*0.001), in the ‘year of diagnosis’ category. In addition, the percentage of patients who received chemotherapy was lower for the PSRCC group, compared with the PDAC group (*p <*0.001). Moreover, in terms of tumor characteristics, larger tumor mass was more common in PSRCC patients than PDAC patients (*p* = 0.001). Additionally, percentage of distant tumors in summary stage (58.1% vs. 43.6%, *p <*0.001) and stage 3 in tumor grade (39.4% vs. 14.1%, *p <*0.001) were higher in PSRCC patients than in PDAC patients. Moreover, there were significant differences in the TNM stages of the two groups (*p <*0.005). However, some variables, such as primary site, LNR, LODDS, surgery/radiation sequence therapy, radiation therapy, sequence number, total number of tumors, race, marital status, and median household income, were similar between the two groups.

**Table 1:** Demographic and clinical characteristics of pancreatic signet ring cell carcinoma (PSRCC) and pancreatic ductal cell carcinoma (PDAC).

Subject	Before PSM		P-value	After PSM		P-value	SD
Characteristic	PDAC	PSRCC		PDAC	PSRCC		
	N (%)	N (%)		N (%)	N (%)		
All	84857	432		431	431		
Year of diagnosis							
(2004,2009]	18777 (22.1)	103 (23.8)	<0.001	98 (22.7)	103 (23.9)	0.7	0.058
<=2004	14752 (17.4)	116 (26.9)		126 (29.2)	115 (26.7)		
>2009	51328 (60.5)	213 (49.3)		207 (48.0)	213 (49.4)		
Primary Site							
250	56865 (67.0)	283 (65.5)	0.134	286 (66.4)	283 (65.7)	0.295	0.131
251	13546 (16.0)	59 (13.7)		66 (15.3)	59 (13.7)		
252	13795 (16.3)	87 (20.1)		79 (18.3)	86 (20.0)		
253	651 (0.8)	3 (0.7)		0 (0.0)	3 (0.7)		
LNR							
(0.1,0.6]	7968 (9.4)	37 (8.6)	0.901	44 (10.2)	37 (8.6)	0.827	0.064
<=0.1	10096 (11.9)	49 (11.3)		48 (11.1)	49 (11.4)		
>0.6	1796 (2.1)	10 (2.3)		12 (2.8)	10 (2.3)		
Unknown	64997 (76.6)	336 (77.8)		327 (75.9)	335 (77.7)		
LODDS							
(-1.1,0.2]	12364 (14.6)	58 (13.4)	0.879	64 (14.8)	58 (13.5)	0.902	0.052
<=-1.1	5759 (6.8)	28 (6.5)		28 (6.5)	28 (6.5)		
>0.2	1737 (2.0)	10 (2.3)		12 (2.8)	10 (2.3)		
Unknown	64997 (76.6)	336 (77.8)		327 (75.9)	335 (77.7)		
Surgery/radiation sequence therapy							
No radiation and/or surgery	77766 (91.6)	400 (92.6)	0.533	393 (91.2)	399 (92.6)	0.533	0.051
Yes	7091 (8.4)	32 (7.4)		38 (8.8)	32 (7.4)		
Radiation							
No/Unknown	69311 (81.7)	369 (85.4)	0.052	367 (85.2)	368 (85.4)	1	0.007
Yes	15546 (18.3)	63 (14.6)		64 (14.8)	63 (14.6)		
Chemotherapy							
No/Unknown	36378 (42.9)	226 (52.3)	<0.001	225 (52.2)	225 (52.2)	1	<0.001
Yes	48479 (57.1)	206 (47.7)		206 (47.8)	206 (47.8)		
Tumor size							
(35,45]	17607 (20.7)	80 (18.5)	0.001	69 (16.0)	80 (18.6)	0.771	0.072
<=35	34699 (40.9)	150 (34.7)		156 (36.2)	150 (34.8)		
>45	19705 (23.2)	110 (25.5)		109 (25.3)	110 (25.5)		
Unknown	12846 (15.1)	92 (21.3)		97 (22.5)	91 (21.1)		
Sequence number							
1st of 2 or more primaries	2026 (2.4)	8 (1.9)	0.569	14 (3.2)	8 (1.9)	0.28	0.088
One primary only	82831 (97.6)	424 (98.1)		417 (96.8)	423 (98.1)		
Total number of tumors							
1	83159 (98.0)	427 (98.8)	0.577	423 (98.1)	426 (98.8)	NA	0.104
2	1596 (1.9)	4 (0.9)		8 (1.9)	4 (0.9)		
3	87 (0.1)	1 (0.2)		0 (0.0)	1 (0.2)		
4	12 (0.0)	0 (0.0)		0 (0.0)	0 (0.0)		
5	3 (0.0)	0 (0.0)		0 (0.0)	0 (0.0)		
Race							
Black	9918 (11.7)	40 (9.3)	0.286	27 (6.3)	40 (9.3)	0.129	0.138
Other/Unknown	7316 (8.6)	37 (8.6)		29 (6.7)	37 (8.6)		
White	67623 (79.7)	355 (82.2)		375 (87.0)	354 (82.1)		
Marital status							
DSW	22233 (26.2)	116 (26.9)	0.686	109 (25.3)	116 (26.9)	0.483	0.107
Married	47972 (56.5)	245 (56.7)		261 (60.6)	244 (56.6)		
Single	11545 (13.6)	52 (12.0)		49 (11.4)	52 (12.1)		
Unknown	3107 (3.7)	19 (4.4)		12 (2.8)	19 (4.4)		
Median household income							
<50000	11452 (13.5)	58 (13.4)	0.907	46 (10.7)	58 (13.5)	0.293	0.107
>70000	35080 (41.3)	183 (42.4)		202 (46.9)	183 (42.5)		
50000-70000	38325 (45.2)	191 (44.2)		183 (42.5)	190 (44.1)		
Age							
<=57	16363 (19.3)	86 (19.9)	0.018	80 (18.6)	86 (20.0)	0.777	0.048
>=73	30535 (36.0)	128 (29.6)		124 (28.8)	128 (29.7)		
58-72	37959 (44.7)	218 (50.5)		227 (52.7)	217 (50.3)		
Grade							
1	3168 (3.7)	1 (0.2)	<0.001	1 (0.2)	1 (0.2)	0.998	0.024
2	13254 (15.6)	26 (6.0)		25 (5.8)	26 (6.0)		
3	11955 (14.1)	170 (39.4)		166 (38.5)	170 (39.4)		
4	408 (0.5)	9 (2.1)		8 (1.9)	8 (1.9)		
Unknown	56072 (66.1)	226 (52.3)		231 (53.6)	226 (52.4)		
Summary stage							
Distant	37009 (43.6)	251 (58.1)	<0.001	249 (57.8)	250 (58.0)	0.904	0.051
Localized	5941 (7.0)	11 (2.5)		14 (3.2)	11 (2.6)		
Regional	27876 (32.9)	126 (29.2)		128 (29.7)	126 (29.2)		
Unknown	14031 (16.5)	44 (10.2)		40 (9.3)	44 (10.2)		
T							
T0/Ti/TX	21472 (25.3)	146 (33.8)	0.001	145 (33.6)	145 (33.6)	0.919	0.066
T1	2768 (3.3)	12 (2.8)		10 (2.3)	12 (2.8)		
T2	17551 (20.7)	67 (15.5)		74 (17.2)	67 (15.5)		
T3	29635 (34.9)	141 (32.6)		143 (33.2)	141 (32.7)		
T4	13431 (15.8)	66 (15.3)		59 (13.7)	66 (15.3)		
N							
N0	36735 (43.3)	153 (35.4)	0.002	151 (35.0)	153 (35.5)	0.936	0.044
N1	23904 (28.2)	123 (28.5)		124 (28.8)	123 (28.5)		
N2	693 (0.8)	4 (0.9)		6 (1.4)	4 (0.9)		
NX	23525 (27.7)	152 (35.2)		150 (34.8)	151 (35.0)		
M							
M0	37152 (43.8)	138 (31.9)	<0.001	149 (34.6)	138 (32.0)	0.574	0.072
M1	34192 (40.3)	192 (44.4)		176 (40.8)	191 (44.3)		
MX	13513 (15.9)	102 (23.6)		106 (24.6)	102 (23.7)		
Sex							
Female	40962 (48.3)	174 (40.3)	0.001	172 (39.9)	174 (40.4)	0.945	0.009
Male	43895 (51.7)	258 (59.7)		259 (60.1)	257 (59.6)		

### 3.2 PSM and survival analysis

PSM was used to balance the baseline clinical characteristics between the PDAC and PSRCC groups (all standard deviations ≤0.05; **Table 1**), and a total of 862 patients (431 PSRCC and 431 PDAC patients) were included in this study after PSM analysis.

**Figure 3:**
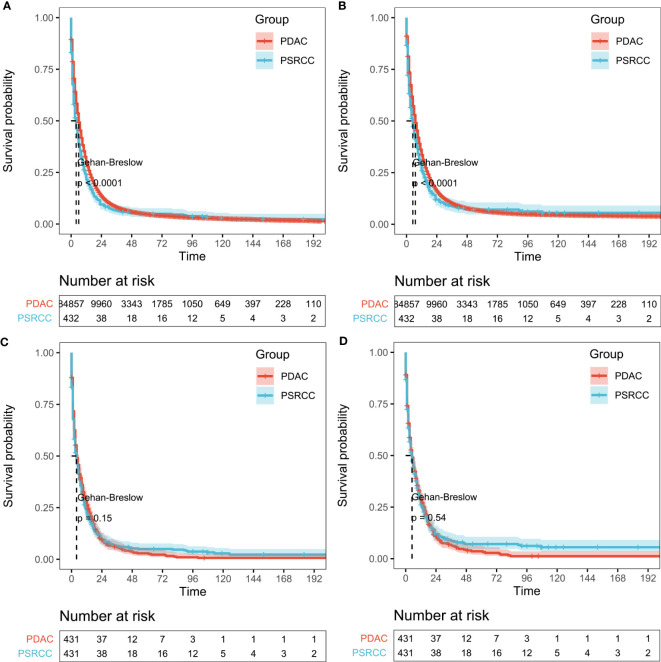
Survival outcomes before and after propensity score matching (PSM). **(A, B)** Overall survival (OS; **(A)** and cancer-specific survival (CSS; **(B)** of pancreatic signet ring cell carcinoma (PSRCC) and pancreatic ductal cell carcinoma (PDAC) patients before PSM and **(C, D)** OS **(C)** and CSS **(D)** of PSRCC and PDAC patients after PSM. Gehan–Breslow tests were used to generate the *P*-values.

After PSM analysis, the PSRCC and PDAC patients were subjected to KM survival analysis. Before PSM, a total of 85289 patients were enrolled in the analysis, and the median OS was 3 and 6 months, while the median CSS was 4 and 7 months for the PSRCC and PDAC groups, respectively. Poorer outcomes were observed in the PSRCC group, compared with the PDAC group, with 1-, 3-, and 5-year OS rates of 21.60% vs. 30.10%, 6.35% vs. 8.12%, and 4.95% vs. 4.76%, respectively, and 1-, 3-, and 5-year CSS rates of 25.97% vs. 33.59%, 8.60% vs. 10.11%, and 7.10% vs. 6.47%, respectively, *p <*0.001; **Figures 3A, B**). However, the post-PSM OS and CSS of the PSRCC and PDAC groups were inconsistent with these results. After PSM, the median OS was 3 and 4 months, while the median CSS was 4 and 5 months for the PSRCC and PDAC groups, respectively. Additionally, the OS and CSS rates were similar between the two groups, with the 1-, 3-, and 5-year OS rates of 21.65% vs. 26.32%, 6.37% vs. 5.74%, and 4.96% vs. 2.90%, *p* = 0.15 and 1-, 3-, and 5-year CSS rates of 26.03% vs. 28.62%, 8.62% vs. 6.48%, and 7.11% vs. 3.60%, respectively, *p* = 0.54; **Figures 3C, D**). Although the prognosis of PDAC patients before PSM seems to be better than that of PSRCC patients, the post-PSM results reveal that there was no significant difference in the prognosis of the two pathological types, after excluding the influence of demographic information, tumor characteristics, and treatment information.

### 3.3 Univariate and multivariate analysis

**Figure 4:**
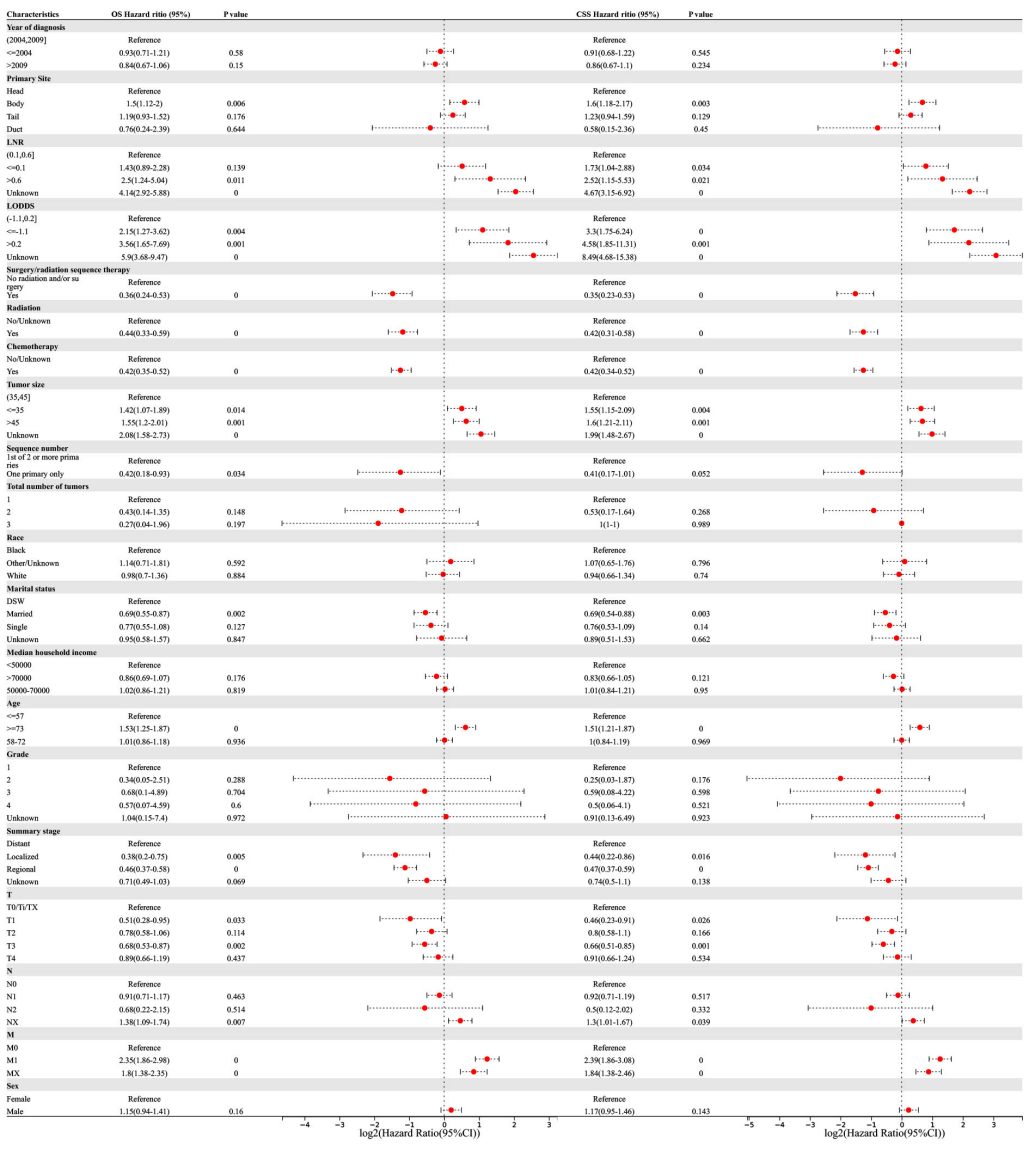
Univariable Cox regression for analyzing overall survival (OS) and cancer-specific survival (CSS) as prognostic factors for pancreatic signet ring cell carcinoma.

Univariate and multivariate Cox regression analyses were performed to determine the potential clinical characteristics which may influence the prognosis of PSRCC patients. Univariate regression analysis revealed that the primary site, LNR, LODDS, surgery/radiation sequence therapy, radiation therapy, chemotherapy, tumor size, marital status, age, summary stage, and TNM stage were prognostic risk factors for both the OS and CSS of PSRCC patients. Additionally, the sequence number was a prognostic factor for the OS of PSRCC patients (*p <*0.05) (**Figure 4**).

**Figure 5:**
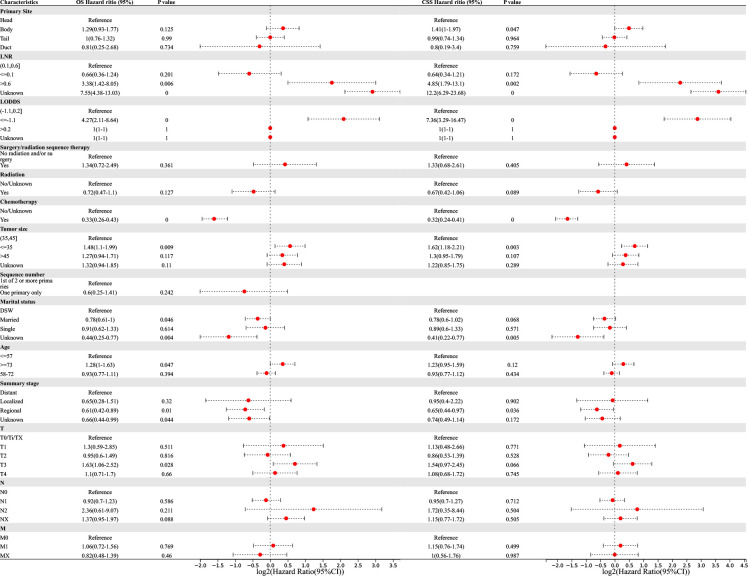
Multivariable Cox regression for analyzing overall survival (OS) or cancer-specific survival (CSS) as prognostic factors for pancreatic signet ring cell carcinoma.

Multivariate regression analysis revealed that higher LNR (‘>0.6’ vs. ‘0.1–0.6’, OR (95% CI) 3.38(1.42–8.05), *p* = 0.006), lower LODDS (‘≤-1.1 L vs. ‘-1.1–0.2’, OR (95% CI) 4.27(2.11–8.64), *p <*0.001), smaller tumor size (‘≤35 vs. ‘35–45’, OR (95% CI) 1.48(1.1–1.99), *p* = 0.009), older age (‘≥73’ vs. ‘≤57’, OR (95% CI) 1.28(1–1.63), *p* = 0.047), and higher T-stage (‘T3’ vs. ‘T0/Ti/TX’, OR (95% CI) 1.63(1.06–2.52), *p* = 0.028) were independent risk factors associated with the OS of PSRCC patients. Additionally, chemotherapy (‘yes’ vs. ‘no/unknown’, OR (95% CI) 0.33(0.26–0.43), *p <*0.001), marital status (‘married’ vs. ‘DSW’, OR (95% CI) 0.78(0.61–1), *p* = 0.046), and regional tumor (‘regional’ vs. ‘distant’, OR (95% CI) 0.61(0.42–0.89), *p* = 0.01) were determined as independent protective factors associated with the OS of PSRCC patients (**Figure 5**).

Meanwhile, pancreatic body cancer (‘body’ vs. ‘head’, OR(95% CI) 1.41(1–1.97), *p* =0.047), higher LNR (‘>0.6’ vs. ‘0.1–0.6’, OR (95% CI) 4.85(1.79–13.1), *p* = 0.002), lower LODDS (‘≤-1.1’ vs. ‘-1.1–0.2’, OR (95% CI) 7.36(3.29–16.47), *p <*0.001), and smaller tumor size (‘≤35’ vs. ‘35–45’, OR (95% CI) 1.62(1.18–2.21), *p* = 0.003) were determined as independent risk factors associated with the CSS of PSRCC patients. Additionally, chemotherapy (‘yes’ vs. ‘no/unknown’, OR (95% CI) 0.32(0.24–0.41), *p <*0.001) and regional tumor (‘regional’ vs. ‘distant’, OR (95% CI) 0.65(0.44–0.97), *p* = 0.036) were determined as independent protective factors associated with the CSS of PSRCC patients (**Figure 5**).

### 3.4 Construction of the OS and CSS predictive models for PDAC and PSRCC

**Figure 6:**
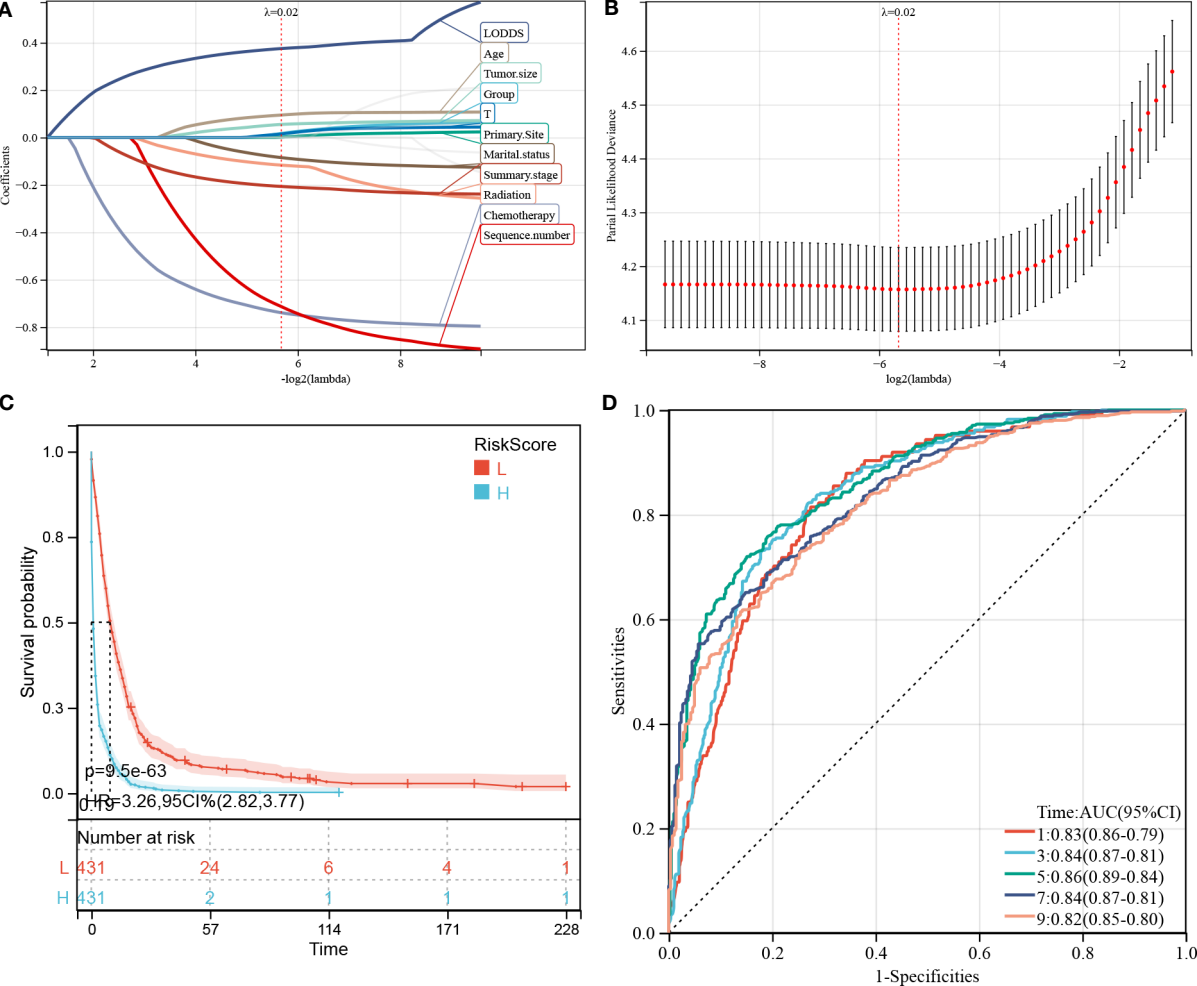
Construction and evaluation of overall survival (OS)-associated predictive models. **(A, B)** The least absolute shrinkage and selection operator (LASSO) coefficient and LASSO deviance profiles. **(C)** Kaplan–Meier analysis of the OS of the high- and low-score groups, according to the prediction model. **(D)** Receiver operating characteristic curves of OS at 1-, 3-, 5-, 7-, and 9-years according to the risk score in the predictive model data sets.

**Figure 7:**
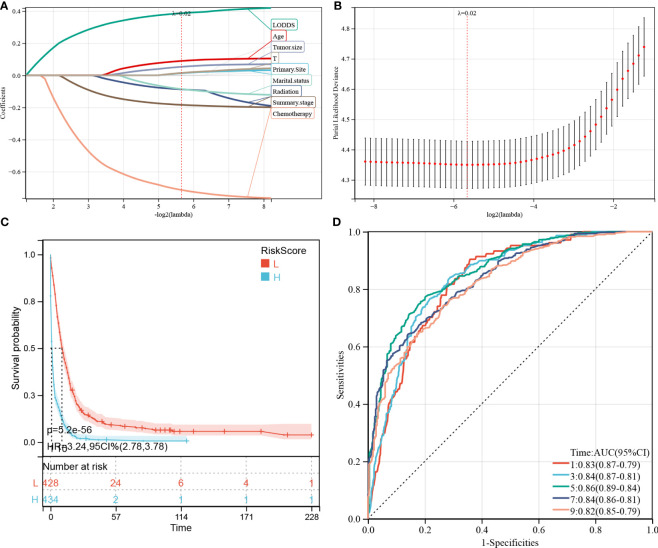
Construction and evaluation of cancer-specific survival (CSS)-associated predictive models. **(A, B)** The least absolute shrinkage and selection operator (LASSO) coefficient **(A)** and LASSO deviance profiles **(B)**. **(C)** Kaplan–Meier analysis of the OS of the high- and low-score groups, according to the prediction model. **(D)** Receiver operating characteristic curves of CSS at 1-, 3-, 5-, 7-, and 9-years according to the risk score in the predictive model data sets.

We performed LASSO regression analysis to construct predictive PDAC/PSRCC OS and CSS models based on the above-mentioned prognostic factors screened by univariate analysis. The predictive models of OS (**Figures 6A, B**) and CSS (**Figures 7A, B**) were constructed by integrating the significant prognostic factors and group information. After 10-fold cross-validation, the optimal λ values of 0.0195 and 0.0198 were obtained for the OS and CSS models, respectively. Finally, 11 prognostic factors, including LODDS, age, tumor size, group, T-stage, primary site, marital status, summary stage, radiation therapy, chemotherapy, and sequence number, were determined for the OS predictive model (**Figure 6A**).

Furthermore, 862 patients screened by PSM were subjected to survival analysis according to the risk score and the optimal cutoff value was determined as -0.586 for the OS model. Thereafter, the patients were classified into high- and low-risk groups, based on the optimal cutoff value. KM curve analysis of the two groups revealed that the OS model could predict patients with good or bad prognoses. The high-risk group manifested a shorter OS than the low-risk group (HR (95% CI) 3.41(2.90–4.02), *p* = 3.1e-54; **Figure 6C**). Similarly, based on the median risk score, the patients could be divided into high- and low-score groups, and survival analysis revealed that the OS of the high-score group is shorter than that of the low-score group (HR (95% CI) 3.26(2.82–3.77), *p* = 9.5e-63). Time-dependent ROC analysis showed that the AUC values of the risk scores for the 1-, 3-, 5-, 7-, and 9-year OS predictions were 0.83, 0.84, 0.86, 0.84, and 0.82, respectively (**Figure 6D**).

Meanwhile, 9 prognostic factors, including LODDS, age, tumor size, T-stage, primary site, marital status, radiation therapy, summary stage, and chemotherapy, were determined for the CSS predictive model (**Figure 7A**). Interestingly, the factors included in the OS prediction model were also included in the CSS prediction model. Similarly, in the KM analysis (**Figure 7C**), the high-risk group manifested a shorter CSS than the low-risk group, based on the optimal cutoff value (3.616; HR (95% CI) 3.23(2.72–3.85), *p* = 7.9e-44) or the median risk score (HR (95% CI) 3.24(2.78–3.78), *p* = 5.2e-56). In the CSS predictive model, the AUC values of the risk scores for predicting 1-, 3-, 5-, 7-, and 9-year CSS were 0.83, 0.84, 0.86, 0.84, and 0.82, respectively (**Figure 7D**).

### 3.5 Validation and visualization of the OS and CSS predictive models

**Figure 8:**
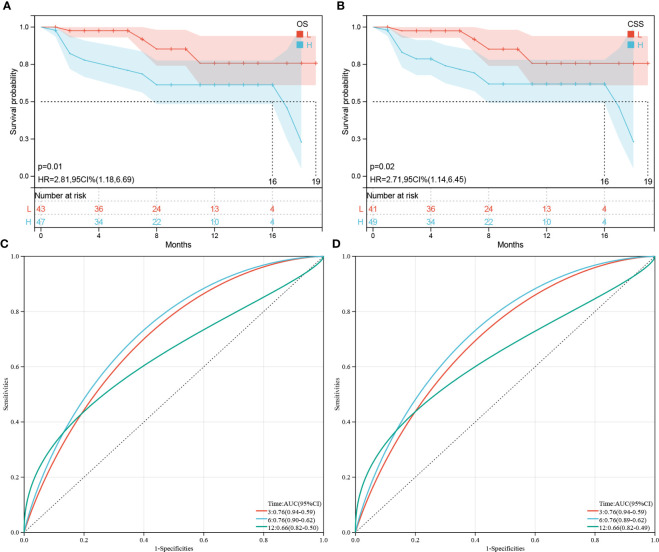
Validation of OS or CSS-associated predictive models. **(A, B)** Kaplan–Meier analysis of the OS and CSS of the high(H)- and low(L)-score groups, respectively, according to the external validation data sets. **(C, D)** Receiver operating characteristic curves of OS and CSS at 3-, 6-, and 12-months according to the risk score in the external validation data sets, respectively.

To verify the prognostic performance of the predictive models, the external data which included 90 patients with PDAC or PSRCC from the Second Xiangya Hospital were used as the validation datasets. **Figures 8A, B** show the KM survival curves of OS and CSS in external validation datasets, respectively. The OS and CSS survival outcomes of patients were significantly different (OS *p* = 0.01, CSS *p* = 0.02). ROC curves were used to evaluate the sensitivity and specificity of the risk score models for the prognoses of patients in the external validation datasets. The results showed that areas under the curve (AUCs) of OS were 0.76, 0.76, and 0.66 at 3-, 6-, and 12-months, respectively (**Figure 8C**). The AUCs at 3-, 6-, and 12-months in the external datasets were 0.76, 0.76, and 0.66, respectively (**Figure 8D**). However, since there were no patients with survival times exceeding 20 months, the 3- and 5-year AUC cannot be calculated. In general, the AUCs of OS and CSS showed good prognostic ability of the predictive models.

**Figure 9:**
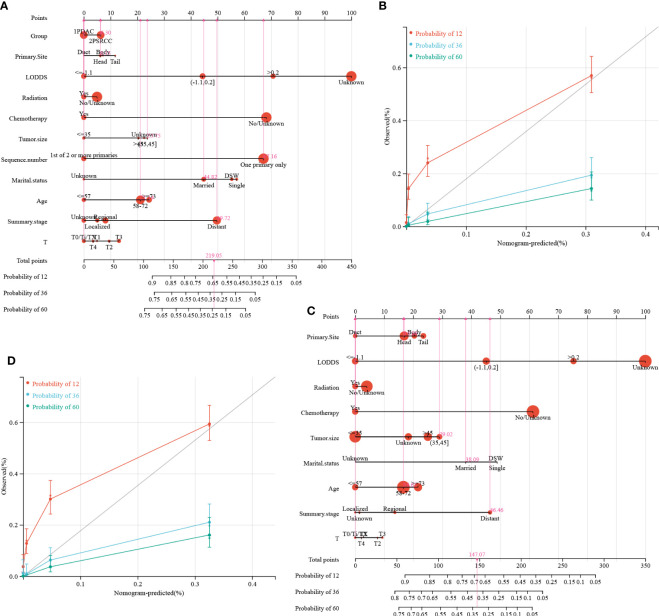
The nomogram of overall survival-associated predictive models. The sum of the scores, indicated by the pink arrows, represents the 1-, 3-, and 5-year survival probability. **(A)** Nomogram for predicting OS in patients with the PSRCC and PDAC patients. **(B)** Calibration curves for 1-year,3-year and 5-year OS in patients with the PSRCC and PDAC patients. **(C)** Nomogram for predicting CSS in patients with the PSRCC and PDAC patients. **(D)** Calibration curves for 1-year, 3-year and 5-year CSS in patients with pancreatic cancer.

Nomogram and calibration curves were used in our study to illustrate the prediction models (**Figures 9A-D**, respectively) and to improve their practicality. The prognostic factors obtained from the LASSO regression analysis were further subjected to COX analysis, and the R package “rms” was used to integrate the survival time, survival status, and characteristic score, to establish the nomogram and draw calibration curves. The perpendicular line from the total point axis to the two-outcome axis allowed us to predict the prognosis of 1-, 3-, and 5-year OS or CSS for the PSRCC and PDAC patients (**Figures 9A, C**). The overall C-indexes of the OS and CSS predictive models were 0.762 (95% CI 0.746–0.779, p = 1.6e-212) and 0.760 (95% CI 0.743–0.777, *p* = 1.3e-188), respectively (**Figures 9B, D**).

## 4 Discussion

PSRCC is a specific type of mucus-secreting adenocarcinoma that is characterized by poor differentiation, diffused invasion, rapid growth, high malignancy, metastasis, and recurrence (16). Primary PSRCC is very rare, accounting for <1% of PCs (17). It primarily occurs in the stomach and accounts for 15.1–28.2% of primary gastric cancers. In this study, we included a total of 432 PSRCC patients from the SEER database, accounting for 0.509% of all the samples, which is slightly less than the previous estimate.

Similar to the other pathological types of PC, PSRCC is difficult to detect in the early stages. PSRCC in the head of the pancreas is usually symptomatic in extrahepatic biliary obstruction, while PSRCC in the other sites is more difficult to detect (18). Early imaging results of PSRCC are mostly negative, and there are no recognized tumor markers of high sensitivity to aid PSRCC diagnosis. The CT of PSRCC shows local, uneven, and low density regions as well as morphological changes in the pancreas, which are similar to the CT of PDAC (19). However, hematoxylin and eosin staining reveals typical signet ring cells under the microscope and is a classic method for diagnosing PSRCC (8). SRCCs are poorly differentiated mucus-secreting adenocarcinomas that can originate in different organs, and all SRCCs, including PSRCC, have a consistent morphology. Therefore, it is not possible to identify the site of origin for transferred SRCCs by cell morphology alone. However, several studies have revealed that mucin, a family of large glycoproteins, is expressed differently in glandular epithelium and adenocarcinomas of different organs and tissues, which can be used to determine the source of SRCC metastases (20).

SRCCs originating in the digestive system, especially PSRCC, generally have a poorer prognosis. A study by Patel et al. (21) revealed that PSRCC has a lower 5-year OS rate than PDAC (4% vs. 9%) and is more likely to present distant metastases at the time of diagnosis (69.4% vs. 52%) (22).Wu et al. reported that the 5-year survival rate of patients with pancreatic SRCC was only 6.4% (23). In a retrospective study, age, location, stage and treatment modality were found to be independent risk factors for predicting OS and DSS in patients with pancreatic SRCC (21). A study conducted by Nie et al. showed that early diagnosis, surgery and chemotherapy are effective methods to improve the prognosis of PSRCC (10).The stage at diagnosis is significantly correlated with the prognosis of patients, and the later the stage, the worse the prognosis (10). Furthermore, Tracey et al. and Chow et al. reported two PSRCC cases that were unresectable and had with a poor prognosis, in which the patients had survived for only a few weeks (24, 25). Strokes et al. (26) reported a 30-year-old male patient with pancreatic body SRCC who experienced recurrence 2 years post-surgery. In addition, Zhang et al. (27) reported that 4 patients with PSRCC had no chance of radical surgery at the time of presentation, and the survival time after palliative surgery did not exceed 5 months, with an average of 2.8 months. In contrast, clinical observations have shown that SRCC of the ampulla of Vater may have a better prognosis when there is no metastasis during surgery (28). The results of this study show that the median OS and CSS of patients with PSRCC are considerably shorter than those of PDAC, with 3 and 4 months, respectively, which is consistent with the previous studies.

Interestingly, analysis of the post-PSM PSRCC and PDAC data revealed similar survival outcome rates between the two groups, indicating that the shorter survival of PSRCC before PSM may be due to inconsistent baseline data. In the baseline data before PSM, the proportion of PSRCC patients with the tumor body was larger and those who received chemotherapy was smaller, which may be the reason for the poor prognosis of PSRCC patients. Therefore, we further explored the potential prognostic factors of PSRCC and found that LNR, LODDS, tumor size, age, T-stage, marital status, and summary stage were independent prognostic factors for PSRCC. Additionally, features which may be associated with the prognosis of PSRCC patients, such as pathological grouping, primary site, radiation therapy, and serial number, were further included in the predictive model and nomogram. Among these, larger tumors, older age, higher T-stage, and distant spread were recognized as poor prognostic factors in tumors. Interestingly, both LNR and LODDS being too large or too small were considered as risk factors for PSRCC in the multivariate COX analysis. However, according to the existing literature and our nomogram results, lower LNR or LODDS corresponds with better prognosis; therefore, the prognostic role of LNR or LODDS in PSRCC should be further explored. Additionally, good marital status may be beneficial for patient survival, which is consistent with the results of Li in ovarian cancer (11). Moreover, among the primary sites of SRCC, pancreatic ductal SRCC has the best prognosis, followed by the head, body, and tail (worst prognosis) of the pancreas. This may be because PSRCC obstruction at the ductal site is the first to appear, and PSRCC at the tail of the pancreas is rarely obstructed and is the most difficult to find.

Due to the lack of clinical data, there is currently no uniform standard for the treatment of primary pancreatic SRCC. Kaji et al. (29) reported a patient with primary PSRCC and mixed gonadal neuroendocrine tumor, who had developed lymph node and liver metastasis. The patient did not undergo surgery, but was given cisplatin + etoposide and albumin-bound paclitaxel + gemcitabine for chemotherapy and had survived for 18 months. Milan et al. (16) reported the presence of a 4.5 cm (diameter) tumor in the head of the pancreas of a 67-year-old female patient, which was further confirmed as PSRCC by biopsy. After 3 months of treatment with gemcitabine the tumor diameter shrank to 1.5 cm, after which pancreaticoduodenectomy was performed, which improve patient prognosis. Terdal (30) first reported the diagnosis of PSRCC by endoscopic retrograde biopsy in the pancreatic duct. The patient underwent pancreaticoduodenectomy, and the surgical specimen showed infiltration of SRCC at the head of the pancreas without metastasis. However, tumor recurrence was observed after 6 months of treatment. Sjoukje et al. (31) reported that one patient with pancreatic head SRCC underwent pancreaticoduodenectomy and died after 18 months of postoperative follow-up. These results indicate that patients who undergo radical surgery have a better prognosis than those who undergo palliative surgery or those who do not undergo surgery, suggesting that surgery is the most effective treatment for early PSRCC patients without metastasis. There is no evidence that chemotherapy is effective for unresectable PSRCC or postoperative adjuvant therapy and there is limited evidence on whether surgery is effective for primary PSRCC patients with distant metastases. In this study, we found that chemotherapy was not only a prognostic factor, but also an independent protective factor that is significantly associated with prognosis, thus providing evidence on the beneficial effects of chemotherapy for PSRCC patients. Additionally, radiotherapy was also found as a prognostic factor; however, it was not an independent prognostic factor, thus its role in PSRCC prognosis needs to be further confirmed. Surprisingly, the results of this study found that the prognosis of patients who underwent sequential surgery/radiotherapy was very different, with no statistically significant difference compared to those who did not receive treatment. We speculate that the cause of this situation is that most patients who received sequential surgery/radiotherapy treatment underwent palliative surgery, which had limited benefits, thus indicating that palliative surgery performed in PSRCC patients should be carefully considered.

Some limitations of this study should also be noted. Firstly, because this study is a retrospective study, there may be a certain recall bias. Second, the SEER database lacks records of specific types of chemotherapy, times of chemotherapy, and whether or not other targeted agents were received. In addition, information on risk factors such as smoking, chronic pancreatitis, alcohol consumption, high-fat diet is not available in the database. Therefore, in order to validate our findings in the future, we recommend that the global PSRCC database conduct retrospective and prospective studies to obtain more comprehensive data and develop appropriate treatment strategies.

## 5 Conclusions

In this study, we found that prognosis of patients with PSRCC were poorer than PDAC, but after balancing the baseline clinical characteristics of PSRCC and PDAC groups by PSM, there was no statistical difference in the prognosis between the two groups. Furthermore, we found that LNR, LODDS, tumor size, age, T-stage, chemotherapy, marital status, and summary stage were independent prognostic factors for the survival of PSRCC patients. Lastly, we generated predictive models and nomograms based on the prognostic factors to predict the individual survival rates of PSRCC and PDAC patients, which can be used in the clinical settings for PSRCC and PDAC management.

## Data availability statement

The original contributions presented in the study are included in the article/**Supplementary Material**. Further inquiries can be directed to the corresponding authors.

## Ethics statement

The studies involving human participants were reviewed and approved by Medical Ethics Committee of the Second Xiangya Hospital of Central South University. The patients/participants provided their written informed consent to participate in this study.

## Author contributions

Z-jZ and X-xL designed and wrote the paper; Y-pH, Y-xW, HuZ, and HeZ produced the figures; Z-tL, YW, and LX reviewed and revised the manuscript. All authors agree to be accountable for the content of the work. All authors contributed to the article and approved the submitted version.
