# Supplementary figures and images for: Prognosis prediction and comparison between pancreatic signet ring cell carcinoma and pancreatic duct adenocarcinoma: a retrospective observational study
Source: Front Endocrinol (Lausanne). 2023 Jul 17;14:1205594. doi: 10.3389/fendo.2023.1205594 (PMC10390323; doi:10.3389/fendo.2023.1205594)

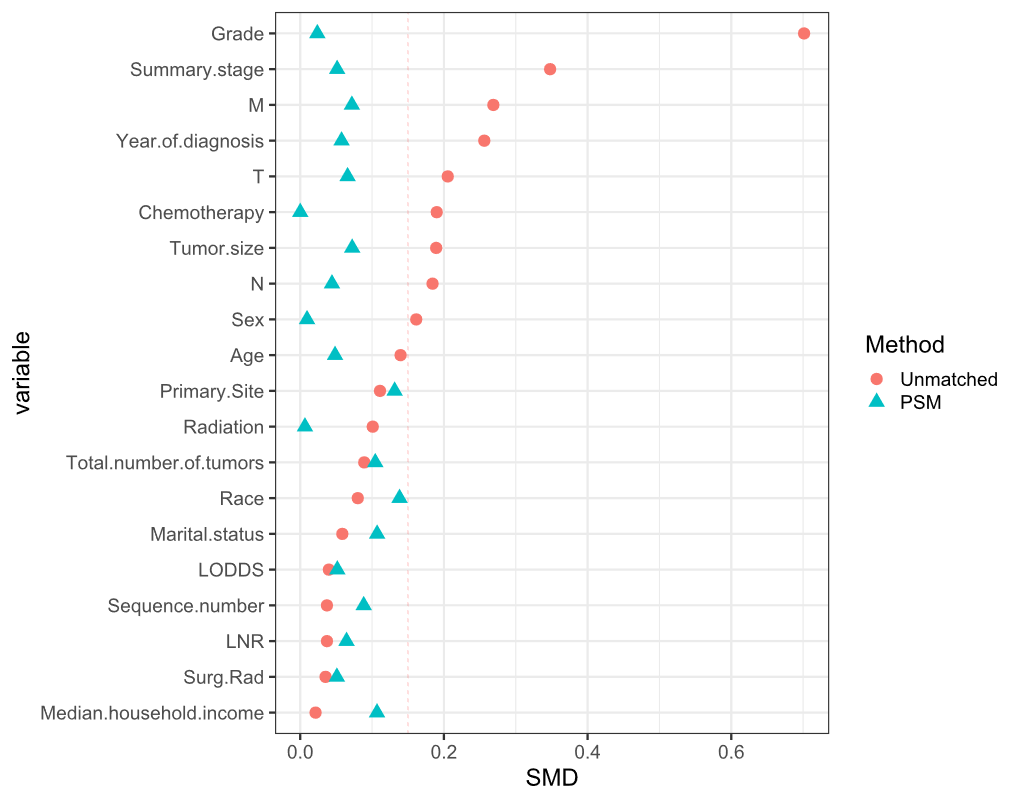

Supplement: Supplementary file 1 [file Image_1.png]

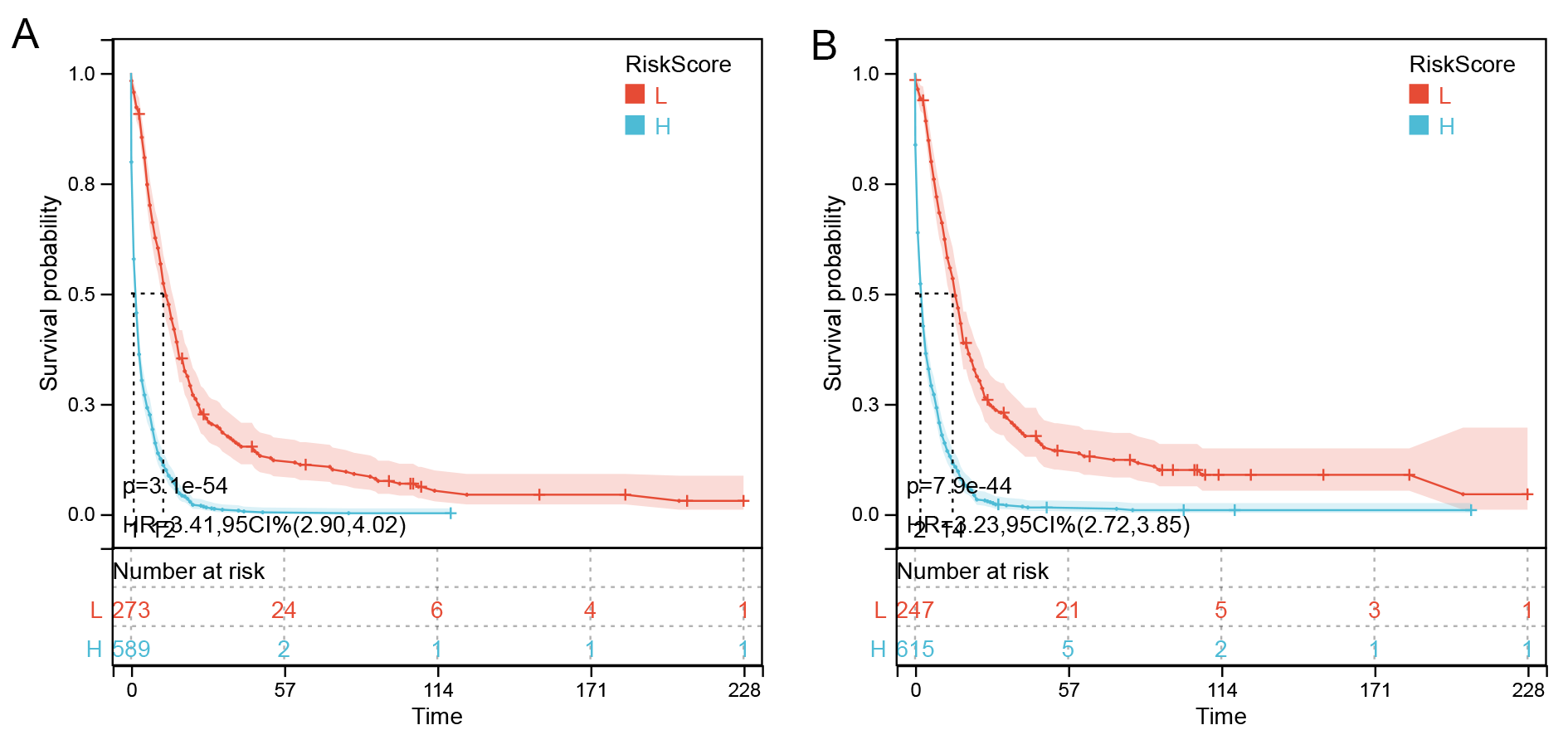

Supplement: Supplementary file 2 [file Image_2.tif]
